# Metagenomics reveals gut microbial differences and ecological adaptation in plateau zokor (*Eospalax baileyi*) populations

**DOI:** 10.1186/s12866-026-05069-6

**Published:** 2026-04-20

**Authors:** Jialong Guo, Chengbo Liang, Lame Cairang, Lei Si, Jingyan Yan, Daoxin Liu

**Affiliations:** 1 College of Agriculture and Animal Husbandry, Qinghai University, Xining, 810016 China; 2 Key Laboratory of Adaptation and Evolution of Plateau Biota, Northwest Institute of Plateau Biology, Chinese Academy of Sciences, Xining, China; 3 College of Life Sciences, University of Chinese Academy of Sciences, Beijing, China; 4 Natural Resources and Forestry & Grassland Bureau of Jianzha County, Huangnan Tibetan Autonomous Prefecture, Qinghai Province, China, Jianzha County, Qinghai Province China; 5 College of Eco–Environmental Engineering, Qinghai University, Xining, 810016 China

**Keywords:** Plateau zokor, Gut microbiome, Metagenomics, Microbiome function, Geographic population

## Abstract

Nine geographically distinct populations of plateau zokors (*Eospalax baileyi*) from Qinghai Province were selected for metagenomic analysis to investigate the composition of gut microbial communities among different populations. The results showed that the core gut microbiota of plateau zokors from different geographic populations was dominated by *Firmicutes*, *Bacteroidetes*, and *Proteobacteria*, with significant differences in community composition among populations. Alpha diversity analysis revealed marked variation in gut microbial diversity and richness across the different geographic populations. Functional prediction further demonstrated significant differences in multiple metabolic pathways, including carbohydrate metabolism, amino acid metabolism, replication and repair, and membrane transport. Notably, carbohydrate-active enzymes associated with the degradation of cellulose, hemicellulose, and lignin exhibited significant differences among populations. In addition, correlation analyses between environmental factors and the gut microbiota indicated that environmental variables such as altitude, annual precipitation, and isothermality had significant effects on gut microbial community structure. Regression analysis between genetic and geographic distances showed that genetic distance among plateau zokor populations increased with increasing geographic distance. Overall, these results suggest that geographic isolation and environmental heterogeneity may jointly drive the differentiation of gut microbial communities in plateau zokors. This study provides microbiological evidence and theoretical support for understanding the ecological adaptation of plateau zokors and offers a scientific basis for the integrated management of grassland rodent pests.

**Supplementary Information:**

The online version contains supplementary material available at 10.1186/s12866-026-05069-6.

## Introduction

The gut microbiota functions as a crucial “microbial organ” within animals, playing key roles in nutrient absorption, immune regulation, energy metabolism, and environmental adaptation, and is essential for maintaining host health and facilitating adaptive evolution [1, 2]. Research has revealed that during animal development, gut microbiota establishes mutualistic relationships with their hosts, and their community diversity typically reflects long-term coevolution between the host and its environment [3, 4]. The composition of gut microbiota is generally shaped by factors such as geographic environment and host species [5]. The gut microbiota of conspecific animals inhabiting different geographic locations often show substantial variation, which is likely driven mainly by differences in environmental factors such as temperature and vegetation type [6]. In wild animals, even within the same species, gut microbiota can exhibit dynamic changes in response to environmental variations during short-term migrations and long-term isolation [7]. Dong et al. [8] found significant differences in the gut microbiota composition of wild Siberian cranes (*Grus leucogeranus*) wintering in different aquatic habitats. Metagenomic analysis by Copeland et al. revealed significant differences in gut microbiota composition and function between second-generation South Asian immigrants in Canada and first-generation immigrants [9]. Likewise, moose populations inhabiting different regions showed substantial variations in both gut microbial diversity and community structure [10]. In-depth understanding of the alienation of intestinal flora in the process of population diffusion and long-term separation is helpful to provide reference for the management of wild animals.

The plateau zokor (*Eospalax baileyi*) is an endemic subterranean rodent of the Qinghai-Tibet Plateau, primarily inhabiting alpine meadows, alpine shrublands, and high-altitude grasslands in the plateau and surrounding regions, at elevations ranging from 2,800 to 4,200 m [11]. The plateau zokor is a typical subterranean rodent that primarily relies on digging tunnels to search for food and establish its habitat. Its diet mainly consists of weedy plants, and it typically feeds on plant roots and rhizomes [12]. Because plateau zokors are subterranean throughout the year, their dispersal is constrained. Their dispersal rate and average distance are markedly lower than those of surface-dwelling animals, and dispersal entails a high energetic cost [13]. At the spatial scale, the patchy distribution of host populations and limited individual dispersal may lead to differentiation of gut microbiota with geographic distance (isolation by distance, IBD). Meanwhile, even over short geographic distances, differences in environmental conditions can also drive microbial community differentiation (isolation by environment, IBE) [14]. Plateau zokors are widely distributed, and different geographic populations exhibit significant variation in spatial patterns, environmental backgrounds, and genetic structures. Environmental factors such as altitude, temperature, and precipitation may also indirectly influence the composition and function of their gut microbiota. However, studies on the gut microbiota of plateau zokors remain limited, particularly those systematically integrating environmental information and host population genetics across multiple geographic populations of the same species. As a result, the relative contributions of geographic isolation, environmental heterogeneity, and host genetic differentiation to gut microbial variation in plateau zokors remain poorly quantified.

Based on this, the present study employed metagenomic sequencing to investigate nine plateau zokor populations distributed across different geographic regions. By combining metagenomic data with environmental and host genetic information, we systematically compared the gut microbiota of different populations in terms of taxonomic composition, diversity, and functional potential, and evaluated the relative roles of geographic distance, environmental differences, and host genetic differentiation in driving microbial divergence. This research aims to elucidate the intrinsic relationships between gut microbiota and the ecological adaptation of plateau zokor populations, providing a theoretical basis for developing region-specific, ecologically friendly, and sustainable management strategies for this species.

## Materials and methods

### Animals

A total of 113 plateau zokor samples were collected from nine geographically distinct populations in Qinghai Province, including Hualong County (HL), Huzhu County (HZ), Datong County (DT), Gonghe County (GH), Gangcha County (GC), Qilian County (QL), Henan County (HN), Maduo County (MD), and Chengduo County (CD). To minimize the potential effects of seasonal variation on the results, all plateau zokors were captured alive between May and June, and the geographic coordinates (latitude and longitude) and elevation of each sampling site were recorded on-site. All plateau zokor samples collected in this study were adult males, as determined based on body size characteristics and reproductive organ morphology observed during field sampling. To avoid potential effects of sex-related hormonal differences on gut microbiome composition, female individuals were not included in this study. Detailed sampling information is provided in Supplementary Tables 1 and Supplementary Fig. 1. In this study, all plateau zokors were deeply anesthetized using carbon dioxide (CO₂) inhalation, and no injectable anesthetic agents were used. Specifically, following capture, the animals were placed in a closed anesthesia chamber and exposed to CO₂ until deep anesthesia was achieved, as confirmed by the complete absence of the pedal withdrawal reflex. After anesthesia induction, euthanasia was performed by cervical dislocation. Upon confirmation of death, abdominal dissection was conducted under sterile conditions to collect cecal content samples. From each individual, one 2-mL cecal content sample was collected, immediately transferred into cryogenic tubes, snap-frozen in liquid nitrogen, and stored for subsequent gut microbial community analyses. The study protocol was reviewed and approved by the Scientific Research Ethics Committee of Qinghai University (IACUC, Approval No.: SL-2023044). All experimental procedures involving plateau zokors were conducted in strict accordance with ethical guidelines for animal research and complied with the standards for the care and use of laboratory animals.

### DNA sequencing and metagenomic sequencing

Total genomic DNA from the gut microbiota of nine geographically distinct plateau zokor populations was extracted using the E.Z.N.A.^®^ Soil DNA Kit (Omega Bio-tek, Norcross, GA, USA). After extraction, DNA concentration and purity were measured, and integrity was checked by 1% agarose gel electrophoresis. Metagenomic sequencing of gut microbiota from nine geographically distinct plateau zokor populations was conducted on the Illumina NovaSeq platform. Genomic DNA was fragmented using a Covaris M220 ultrasonic device (Gene Company, China), and fragments of approximately 350 bp were selected for paired-end (PE) library construction. Paired-end (PE) libraries were prepared using the NEXTFLEX^®^ Rapid DNA-Seq kit (Bioo Scientific, USA) according to the following procedure: (1) ligation of adapters; (2) removal of adapter dimers using magnetic bead-based selection; (3) enrichment of library templates via PCR amplification; and (4) purification of PCR products with magnetic beads to obtain the final libraries. Data analysis started with the raw sequencing data. Initial quality control was performed using fastp (https://github.com/OpenGene/fastp, version 0.20.0) to trim adapter sequences from the 3′ and 5′ ends and remove reads with an average base quality score below 20 or a length shorter than 50 bp. The quality-filtered reads were then aligned to host DNA sequences using BWA (http://bio-bwa.sourceforge.net, version 0.7.17) (host genome accession: GCA_035773235.1), and reads exhibiting high similarity to the host genome or representing potential contaminants were removed, yielding high-quality clean data for downstream analyses [15, 16]. Quality-filtered reads were assembled using MEGAHIT (https://github.com/voutcn/megahit, version 1.1.2), and contigs of at least 300 bp were retained as the final assembly. Open reading frames (ORFs) were predicted from the assembled contigs using Prodigal (https://github.com/hyattpd/Prodigal, version 2.6.3). Genes with nucleotide lengths of 100 bp or longer were selected and translated into amino acid sequences, generating predicted gene sets for each sample [17, 18]. All predicted gene sequences from the samples were clustered using CD-HIT (http://weizhongli-lab.org/cd-hit/, version 4.7) with a 90% sequence identity and 90% coverage threshold. The longest gene in each cluster was chosen as the representative sequence, generating a non-redundant gene set [19]. Finally, high-quality reads from each sample were mapped to the non-redundant gene set using SOAPaligner (https://github.com/ShujiaHuang/SOAPaligner, version SOAP2.21 release) [20].

Genomic DNA was extracted from muscle tissue of plateau zokors using the Ezup Column Animal Genomic DNA Extraction Kit (Sangon Biotech, Shanghai, China) following the manufacturer’s protocol. DNA concentration and purity were measured with a NanoDrop 2000c spectrophotometer (Thermo Scientific). Genomic DNA from two individual plateau zokors was then used for reduced-representation genome sequencing (RAD-seq) on the Illumina HiSeq™ 2500 platform. Simple sequence repeat (SSR) loci in the genome were identified using SR Search software, and a subset of these loci was selected for primer design and PCR amplification. After initial screening and validation of polymorphism, 10 highly polymorphic SSR primer pairs were chosen for genetic distance analysis among plateau zokor populations. PCR products were checked by gel electrophoresis and then submitted to Sangon Biotech (Shanghai, China) for fragment length determination. The PCR amplification conditions were as follows: the total reaction volume was 20 µL, containing 1 µL of template DNA (50 ng/µL), 1 µL of forward primer (10 µM), 1 µL of reverse primer (10 µM), 1 µL of dNTP mixture (10 µM), 2.5 µL of 10× Taq buffer (with MgCl₂), 0.5 µL of Taq DNA polymerase (5 U/µL), and nuclease-free ddH₂O added to a final volume of 20 µL.The PCR cycling program was as follows: an initial denaturation at 95 °C for 3 min; followed by 10 cycles of denaturation at 94 °C for 30 s, annealing at 60 °C for 30 s, and extension at 72 °C for 30 s; then 35 cycles of denaturation at 94 °C for 30 s, annealing at 55 °C for 30 s, and extension at 72 °C for 30 s; with a final extension at 72 °C for 8 min.

### Sequencing data processing

The non-redundant gene set was aligned to the Non-Redundant Protein Sequence (NR) database and the Kyoto Encyclopedia of Genes and Genomes (KEGG) database using Diamond (https://github.com/bbuchfink/diamond, version 2.0.13) with BLASTP parameters set to an e-value threshold of 1e-5. This allowed for taxonomic annotation based on the NR database and functional annotation according to KEGG pathways [21]. The amino acid sequences of the non-redundant gene set were aligned to the Carbohydrate-Active Enzymes (CAZy) database using HMMER (http://hmmer.org/, version 3.1b2) with an e-value cutoff of 1e-5, yielding annotations for genes corresponding to carbohydrate-active enzymes. Because metagenomic data may contain DNA fragments derived from the host’s diet or environmental sources, plant or viral taxa may be detected in the NR database annotation results. This study primarily focused on the structure of the gut-colonizing microbial community. Therefore, in subsequent analyses of community composition and diversity, the analysis mainly focused on bacterial taxa, while plant and viral sequences were not included as core members of the microbial community in comparative analyses.

Differences in gut microbiota among geographic populations of plateau zokors at the phylum, genus, and functional gene levels were analyzed using the Kruskal–Wallis rank-sum test, and the results were adjusted for multiple comparisons using the Benjamini–Hochberg FDR correction, with significance set at FDR ≤ 0.05. α-diversity of the gut microbial communities was calculated using the boot and stats packages in R.

In the β-diversity analysis, β-diversity distance matrices were first calculated using QIIME (version 2020.2.0), and differences between groups were assessed via ANOSIM with 999 permutations. Community dissimilarity among samples was quantified using Binary Hamming distances, and differences in community structure were visualized using principal coordinates analysis (PCoA) and non-metric multidimensional scaling (NMDS).Differential analyses of gut microbial communities and KEGG pathways were performed using LEfSe (http://galaxy.biobakery.org/).Environmental variables were obtained from WorldClim (http://www.worldclim.org/) And extracted for each individual based on GPS coordinates using ArcGIS (version 10.8), resulting in a total of 22 variables. To minimize multicollinearity, pairwise Spearman correlation coefficients among environmental variables were calculated using the varclus function in R package Hmisc (version 4.7-0) with method = “spearman”. Variables with absolute correlation coefficients |ρ| > 0.8 were considered highly correlated, and within each set of highly correlated variables, the representative variable explaining the largest proportion of variance was retained to reduce multicollinearity. db-RDA analysis was performed based on Bray–Curtis distance matrices using the vegan package (version 2.4-3) in R (version 3.3.1).

Genetic distances among the nine geographic populations of plateau zokors were calculated using GenAlEx 6.5, while geographic distance matrices were calculated based on the latitude and longitude of sampling sites. Mantel tests were performed to assess the correlation between geographic and genetic distances using the mantel function in vegan (version 2.4-3) with Pearson correlation and 9,999 permutations. Additionally, the distance–decay relationship between geographic distance and gut microbial community dissimilarity (based on Bray–Curtis distances) was evaluated using linear regression in R (version 3.3.1), and the results were visualized using scatter plots.

## Results

### Characteristics of the metagenomic dataset

**Fig. 1:**
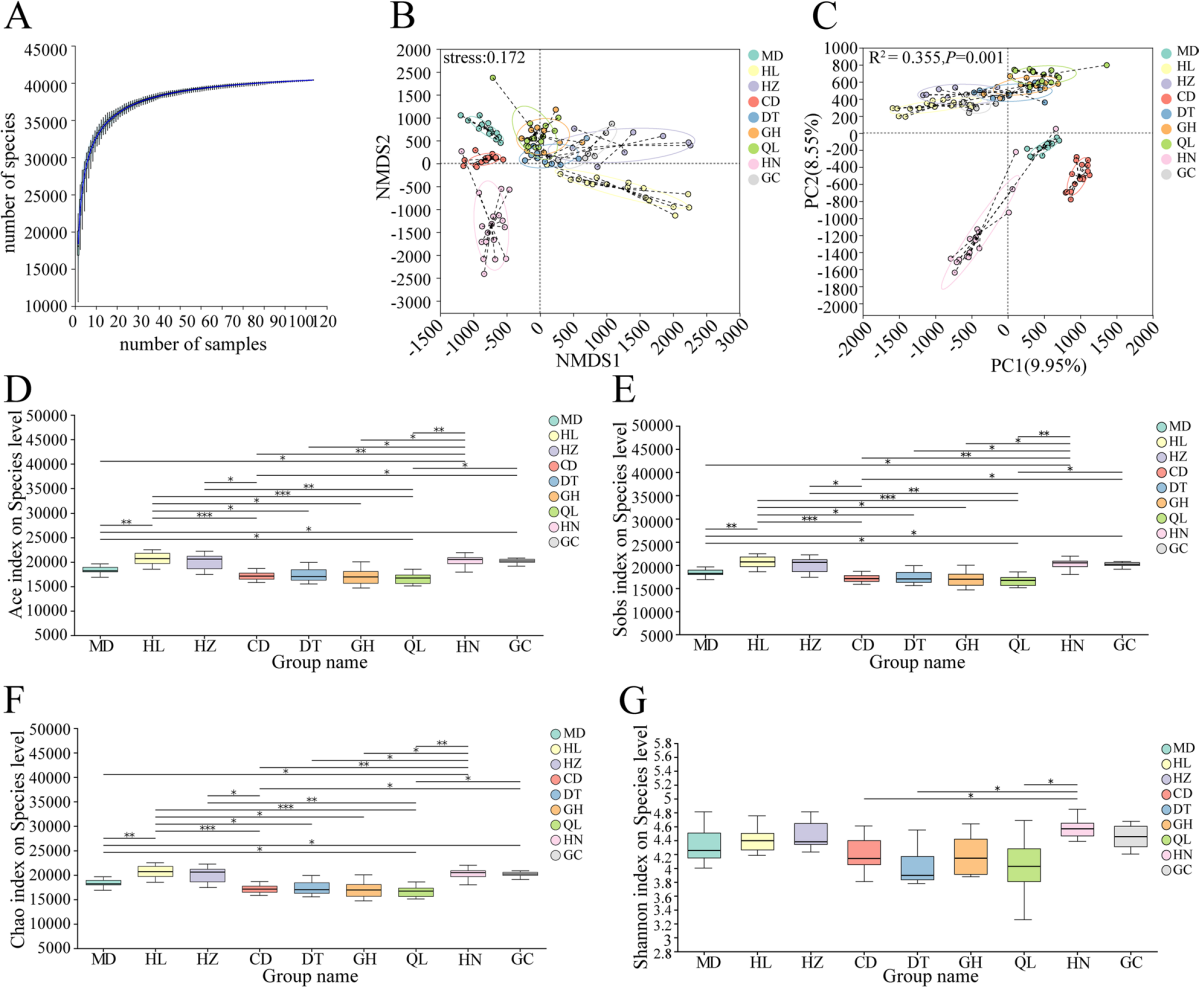
Species accumulation curves and gut microbial community diversity analysis of plateau zokor populations. **A** Species accumulation curves of nine plateau zokor populations. **B** NMDS analysis based on binary Hamming distance (stress = 0.172). **C** PCoA analysis based on binary Hamming distance (R² = 0.355, *P* = 0.001), with different circle colors representing different zokor populations. **D** Comparison of ACE index between groups. **E** Comparison of Sobs index between groups. **F** Comparison of Chao index between groups. **G** Comparison of Shannon index between groups. (* *P* ≤ 0.05, ** *P* ≤ 0.01, *** *P* ≤ 0.001)

A total of 113 samples from various geographic populations of plateau zokors were sequenced, yielding 10,713,819,136 raw reads. After quality control and filtering, an average of 84,158,996 clean reads per population were retained. The average GC content was 47.73%, with Q20 scores ≥ 97.97% and Q30 scores ≥ 94.05% (Supplementary Table 2). Species accumulation curves approached saturation with increasing sequencing depth, indicating that the sequencing depth was sufficient for downstream analyses and that the data were representative (Fig. 1A). The effective reads were assembled and analyzed, and the numbers of contigs and ORFs were averaged across the different geographic populations for statistical evaluation. The contig lengths ranged from 913,657.81–1,267,970.50 bp, with total sequence lengths of 670,313,951.80–923,771,568.60 bp. The longest contigs measured 298,907.40–368,151.25 bp, while the shortest contigs were 300 bp. The N50 values ranged from 771.22–1,259.86 bp, and N90 values ranged from 361.56 to 384.57 bp. Gene prediction identified an average of 1,176,484.93–1,719,523.54 ORFs across populations (Supplementary Table 3). These results indicate that the sequencing was of high quality and depth, providing a robust dataset suitable for subsequent gene annotation and functional analyses.

### Diversity of gut microbiota across different geographical populations of plateau zokors

Alpha diversity of the gut microbiota was assessed for nine geographical populations of plateau zokors. For richness indices including ACE, Sobs, and Chao, the five populations with the highest diversity, in descending order, were HL, GC, HZ, HN, and MD. Pairwise comparisons among all nine populations identified 18 groups showing significant differences (*P* < 0.05) (Fig. 1D – F). Analysis of the Shannon index indicated that the five populations with the highest gut microbiota diversity, in descending order, were HN, HZ, GC, HL, and MD. Significant differences were detected between HN and CD, HN and DT, and HN and QL (*P* < 0.05) (Fig. 1G).

Beta diversity of the gut microbiota was assessed for different geographical populations of plateau zokors. Principal coordinates analysis (PCoA) (Fig. 1C) revealed that individuals from the same population clustered together, with significant differences detected among the nine populations (R² = 0.355, *P* = 0.001). Non-metric multidimensional scaling (NMDS) analysis (Fig. 1B) showed similar clustering patterns, consistent with the PCoA results. Notably, populations MD, CD, and HN exhibited greater differences in gut microbial composition compared to the other populations, indicating distinct gut microbiota structures among the geographical populations.

### Composition and differences of gut microbiota among different geographic populations of plateau zokors

**Fig. 2:**
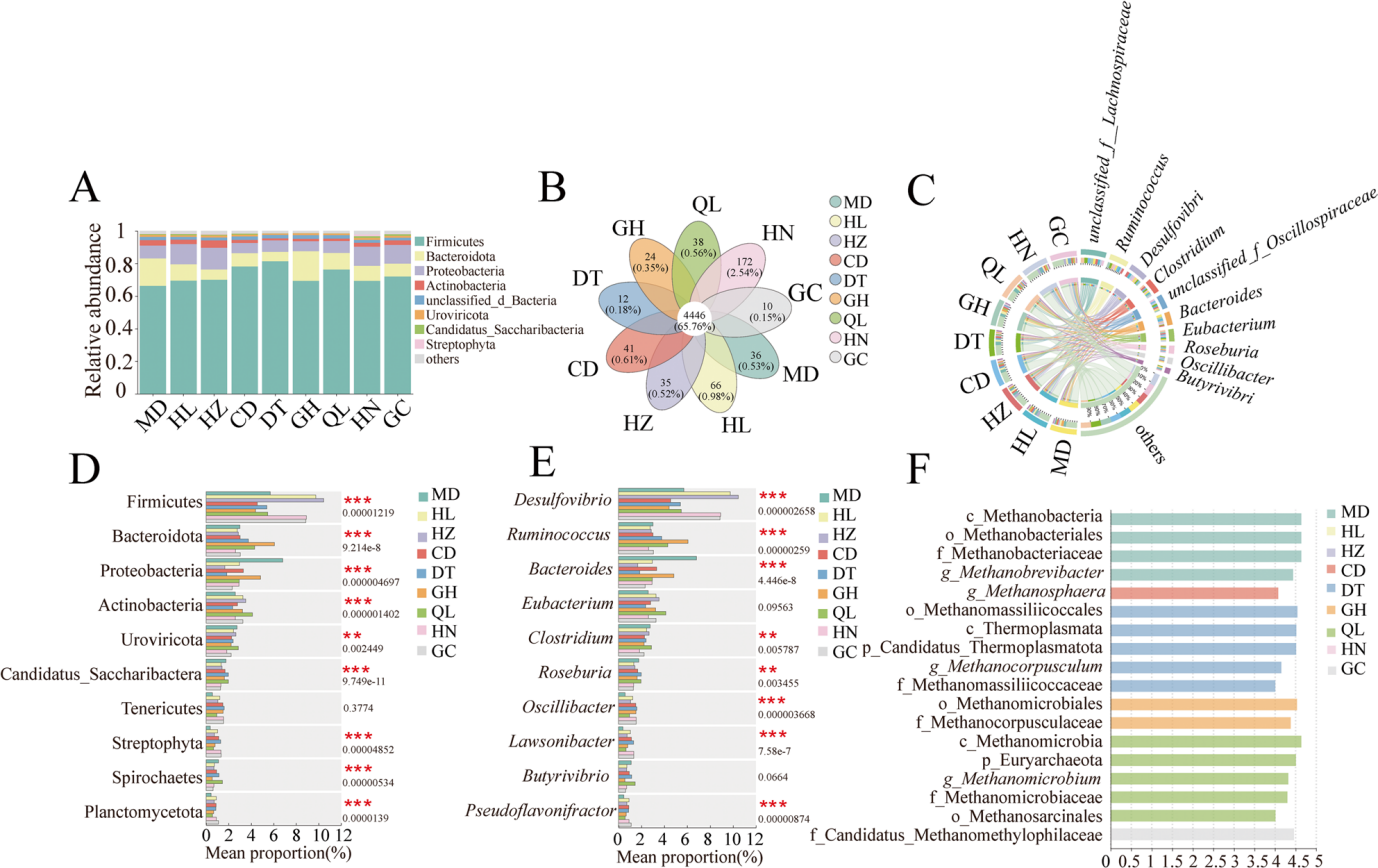
Gut microbial community composition and differential analysis of different geographic populations of plateau zokors. **A** Phylum-level community bar plot of gut microbiota in plateau zokors from different geographic populations. **B** Venn diagram showing shared and unique microbial taxa among plateau zokors from different geographic populations. **C** Genus-level community Circos plot of plateau zokors from different geographic populations. **D** Bar plot of phylum-level differential abundance analysis. **E** Bar plot of genus-level differential abundance analysis. **F** Taxa showing significant differences from phylum to genus level among plateau zokors from different geographic populations under an LDA score threshold of 4. (* *P* ≤ 0.05, ** *P* ≤ 0.01, *** *P* ≤ 0.001)

According to the Venn diagram, the unique or common species (genus level) among different geographical populations of plateau zokor were analyzed (Fig. 2B). The results showed that a total of 4,446 bacterial species were shared among the nine geographical populations of plateau zokors. The number of unique bacterial species in each population was as follows: 66 in HL, 35 in HZ, 12 in DT, 24 in GH, 10 in GC, 38 in QL, 172 in HN, 36 in MD, and 41 in CD.

At the phylum level, the gut microbiota of nine geographical populations of plateau zokors were identified. The results showed that the top three dominant bacterial phyla across all populations were *Firmicutes*, *Bacteroidetes*, and *Proteobacteria* (Fig. 2A). The relative abundances of *Firmicutes* (MD: 67.46%, HL: 70.67%, HZ: 71.31%, CD: 79.85%, DT: 82.93%, GH: 71.38%, QL: 78.23%, HN: 70.63%, GC: 72.96%); *Bacteroidetes* (MD: 17.92%, HL: 10.68%, HZ: 6.92%, CD: 8.93%, DT: 6.53%, GH: 18.54%, QL: 10.67%, HN: 9.79%, GC: 8.70%) and the phylum *Proteobacteria* (MD: 8.36%, HL: 13.07%, HZ: 13.97%, CD: 8.93%, DT: 7.43%, GH: 6.64%, QL: 7.75%, HN: 12.45%, GC: 12.10%). respectively. Although the relative abundances of these bacterial phyla varied among different geographical populations, *Firmicutes*, *Bacteroidetes*, and *Proteobacteria* consistently represented the dominant phyla in the gut microbiota of plateau zokors. At the genus level (Fig. 2C), the top five dominant genera in terms of relative abundance across the nine geographical populations of plateau zokors were as follows, GC group: *Desulfovibrio* (9%), *Ruminococcus* (8%), *an unclassified genus from the Lachnospiraceae family* (7%), an *unclassified genus from the Oscillospiraceae family* (7%), and *Clostridium* (6%). HN group: *Desulfovibrio* (9%), *an unclassified genus from the Lachnospiraceae family* (8%), *Ruminococcus* (7%), *an unclassified genus from the Oscillospiraceae family* (7%), *Clostridium* (5%), and *Bacteroides* (5%). QL group: *an unclassified genus from the Lachnospiraceae family* (14%), *Ruminococcus* (8%), *Clostridium* (7%), *Desulfovibrio* (6%), and *Eubacterium* (6%). GH group: *an unclassified genus from the Lachnospiraceae family* (12%), *Ruminococcus* (10%), *Bacteroides* (8%), *Desulfovibrio* (5%), and *Clostridium*. DT group: *an unclassified genus from the Lachnospiraceae family* (14%), *Ruminococcus* (9%), *an unclassified genus from the Oscillospiraceae family* (7%), *Desulfovibrio* (6%), and *Clostridium* (6%). CD group: *an unclassified genus from the Lachnospiraceae family* (12%), *Ruminococcus* (7%), *an unclassified genus from the Oscillospiraceae family* (7%), *Clostridium* (6%), and *Desulfovibrio* (5%). HZ group: *Desulfovibrio* (11%), *an unclassified genus from the Lachnospiraceae family* (10%), *Ruminococcus* (6%), *Clostridium* (6%), *and an unclassified genus from the Oscillospiraceae family* (6%). HL group: *Desulfovibrio* (10%), *an unclassified genus from the Lachnospiraceae family* (9%), *Ruminococcus* (7%), *Clostridium* (6%), and *an unclassified genus from the Oscillospiraceae family* (6%). MD group: *an unclassified genus from the Lachnospiraceae family* (10%), *Bacteroides* (10%), *Ruminococcus* (8%), *Desulfovibrio* (6%), and *Clostridium* (6%).

At the phylum level, a significance test of the gut microbiota across the nine geographical populations of plateau zokors was performed (Fig. 2D). The results showed that the top ten phyla with significant differences in relative abundance were *Firmicutes*, *Bacteroidetes*, *Proteobacteria*, *Actinobacteria*, *Uroviricota*, *Candidatus Saccharibacteria*, *Streptophyta*, *Spirochaetes*, and *Planctomycetota*. At the genus level (Fig. 2E), the top ten genera with significant differences in relative abundance were *Desulfovibrio*, *Ruminococcus*, *Bacteroides*, *Clostridium*, *Roseburia*, *Oscillibacter*, *Lawsonibacter*, and *Pseudoflavonifractor*. To further identify differential microbial taxa from the phylum to genus levels and pinpoint key gut microbiota among the nine geographical populations of plateau zokors, linear discriminant analysis effect size (LEfSe) was performed. The LDA score threshold was set at 4 (Fig. 2F). A total of 18 microbial biomarkers with significant differences were identified across the nine geographical populations of plateau zokors. Specifically, MD group: Methanobacteria, Methanobacteriales, Methanobacteriaceae, and *Methanobrevibacter*; CD group: *Methanosphaera*; DT group: Methanomassiliicoccales, Thermoplasmata, *Candidatus Thermoplasmatota*, *Methanocorpusculum*, and Methanomassiliicoccaceae; GH group: Methanomicrobiales and Methanocorpusculaceae; QL group: Methanomicrobia, *Euryarchaeota*, *Methanomicrobium*, Methanomicrobiaceae, and Methanosarcinales; GC group: Candidatus Methanomethylophilaceae.

### Functional analysis of the gut microbiota in plateau zokors

**Fig. 3:**
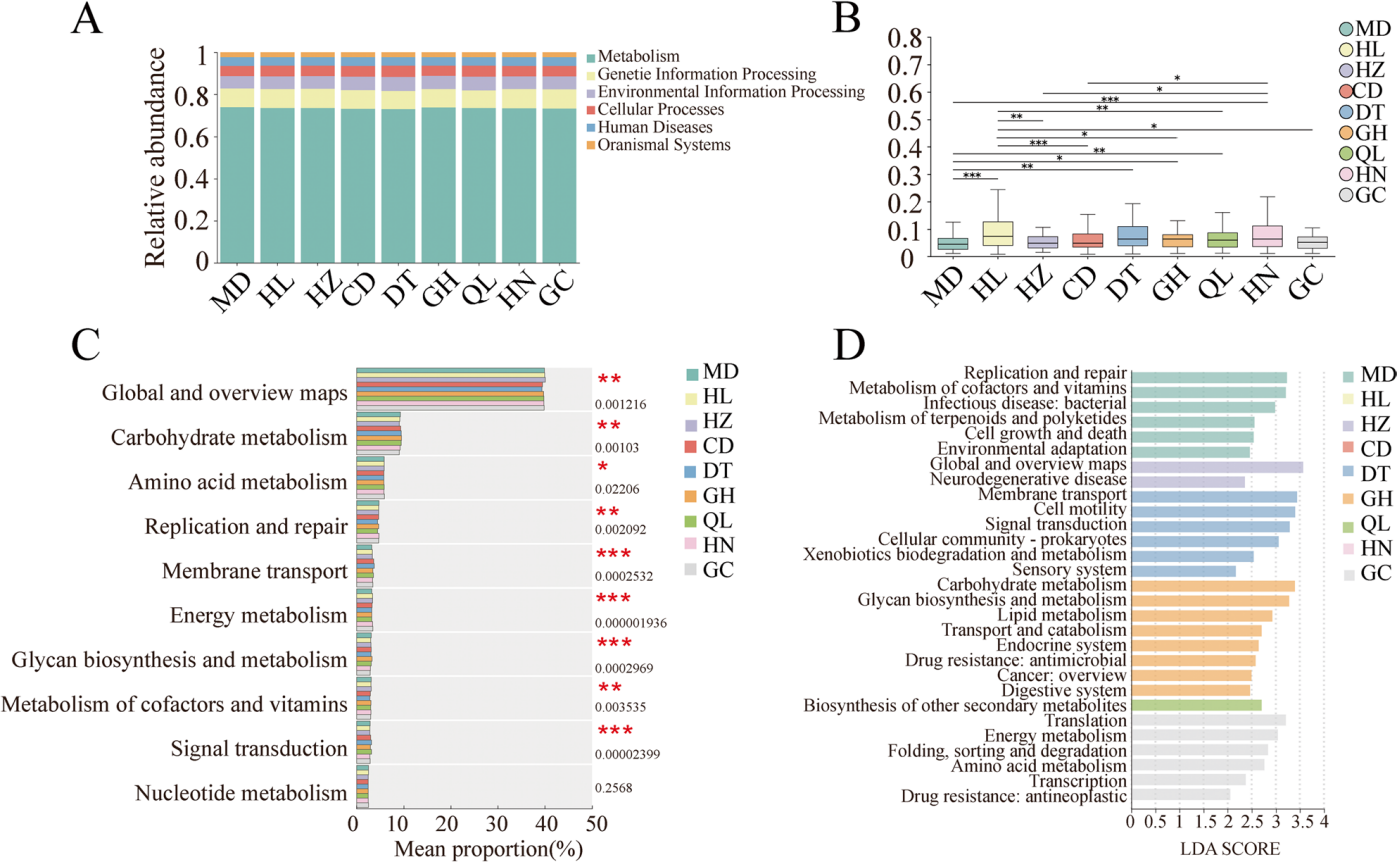
KEGG functional annotation of gut microbiota in different plateau zokor populations. **A** Level 1 KEGG functional annotation relative abundance bar chart. **B** Level 2 KEGG functional annotation inter-group significance test bar chart. **C** Level 2 KEGG functional annotation differential abundance bar chart (* *P* ≤ 0.05, ** *P* ≤ 0.01, *** *P* ≤ 0.001). **D** Level 2 KEGG functional annotation LDA analysis of plateau zokors from different geographic populations (LDA score threshold ≥ 4)

The functional genes of gut microbiota from different geographical populations of plateau zokors were annotated using the KEGG (Kyoto Encyclopedia of Genes and Genomes) database. At Level 1, the annotated genes were classified into six functional categories. Across all populations, the relative abundances of these categories ranked from highest to lowest as follows: Metabolism, Genetic Information Processing, Environmental Information Processing, Cellular Processes, Organismal Systems, and Human Diseases. The majority of genes in the gut microbiota were associated with metabolic functions, followed by genetic information processing and environmental information processing (Fig. 3A). At Level 2, the gut microbiota of plateau zokors exhibited the highest relative abundance in the “Global and overview maps” category (40.51% ± 0.23%), followed by Carbohydrate Metabolism (9.18% ± 0.16%) and Amino Acid Metabolism (5.80% ± 0.04%) (Supplementary Table 4).

Significance testing of gut microbiota functions at KEGG Level 2 across different geographical populations of plateau zokors revealed 12 pairs of intergroup comparisons with significant differences (*P* < 0.05) (Fig. 3B). Among these, the top five metabolic pathways showing the most significant differences were Global and Overview Maps, Carbohydrate Metabolism, Amino Acid Metabolism, Replication and Repair, and Membrane Transport (Fig. 3C). In the linear discriminant analysis (LDA), with an LDA threshold set at 2, the distribution of LDA scores is presented as a bar plot (Fig. 3D). MD group: Replication and Repair, Metabolism of Cofactors and Vitamins, Infectious Disease: Bacterial, Metabolism of Terpenoids and Polyketides, Cell Growth and Death, and Environmental Adaptation. HZ group: Global and Overview Maps, and neurodegenerative disease. DT group: Membrane Transport, Cell Motility, Signal Transduction, Cellular Community – Prokaryotes, Xenobiotics Biodegradation and Metabolism, and Sensory System. GH group: Carbohydrate Metabolism, Glycan Biosynthesis and Metabolism, Lipid Metabolism, Transport and Catabolism, Endocrine System, Drug Resistance: Antimicrobial, Cancer: Overview, and Digestive System. QL group: Biosynthesis of Other Secondary Metabolites. GC group: Translation, Energy Metabolism, Folding, Sorting and Degradation, Amino Acid Metabolism, Transcription, and Drug Resistance: Antineoplastic.

### CAZy analysis of gut microbiota in different plateau zokor populations

**Fig. 4:**
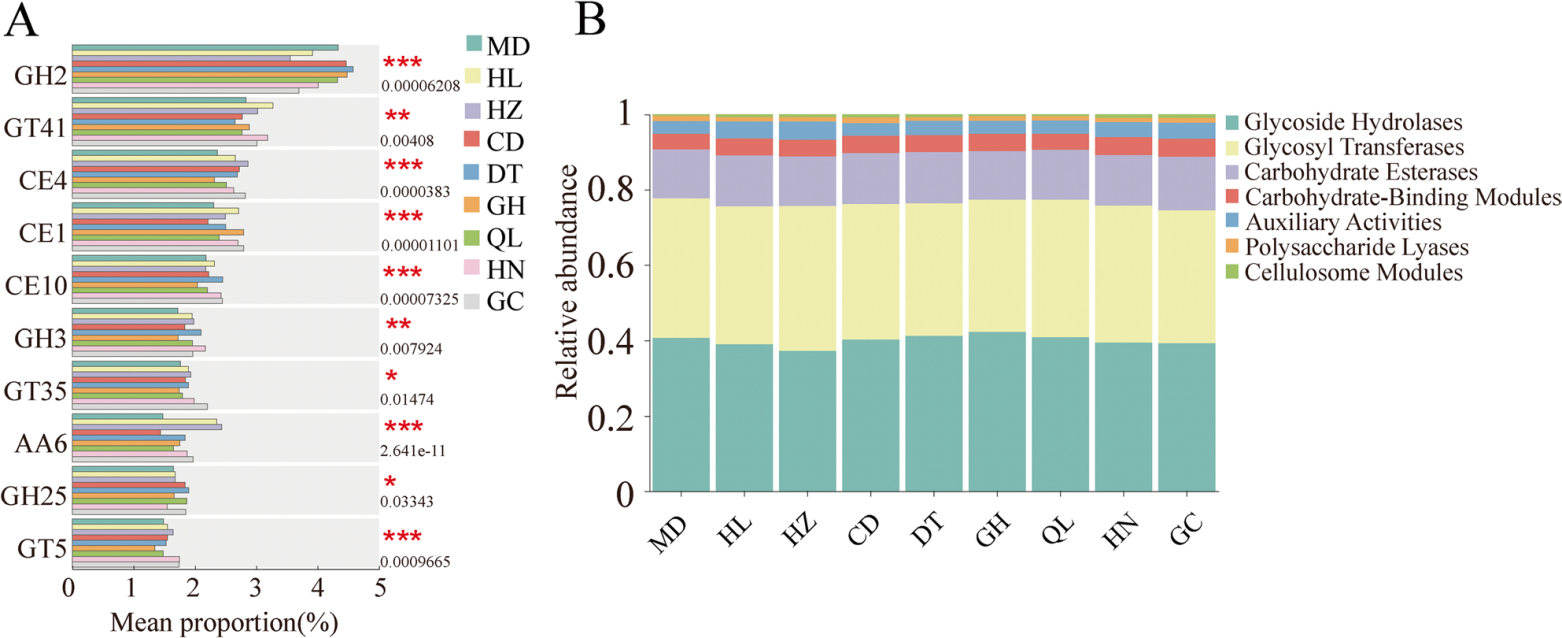
Composition and differences of gut microbial CAZymes in different geographic populations of plateau zokors. **A** Differential analysis of CAZyme families in the gut microbiota of different geographic populations of plateau zokors (rank-sum test). **B** Relative abundance bar chart of CAZymes functional classes (class level) in the gut microbiota of different geographic populations, with colors representing different enzyme classes

The functional genes of different plateau zokor populations were compared against the Carbohydrate-Active Enzymes (CAZy) database. At the class level, the CAZy annotation identified a total of seven types of carbohydrate-active enzyme genes. Among these, glycoside hydrolases accounted for the highest proportion, followed by glycosyltransferases, carbohydrate esterases, carbohydrate-binding modules, auxiliary activities, polysaccharide lyases, and cellulosome modules (Fig. 4B). To further explore the differences in carbohydrate-active enzyme genes among gut microbiota of different plateau zokor populations, intergroup comparisons were performed at the CAZy family level. The results showed that the main differences among populations were in glycoside hydrolases (GH), glycosyltransferases (GT), and carbohydrate esterases (CE) gene families. Specifically, the relative abundances of GH2, GT41, CE4, CE1, CE10, GH3, GT35, and AA6 exhibited significant differences among the various plateau zokor populations (Fig. 4A).

### Analysis of the effects of geographical distance, environmental factors, and genetic differentiation on gut microbiota of different plateau zokor populations

**Fig. 5:**
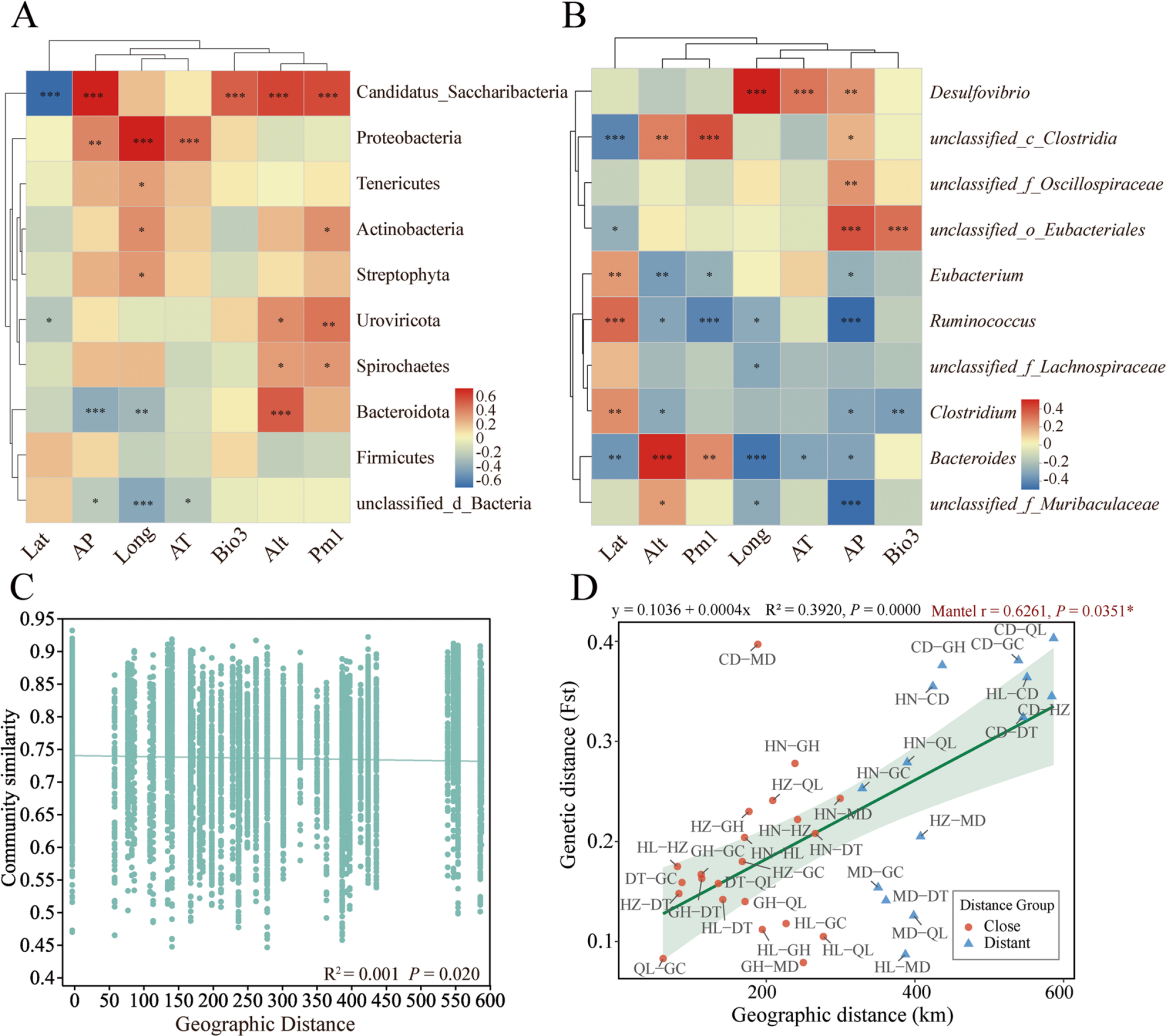
Analysis of gut microbiota in relation to environmental factors and genetic distance. **A** Correlation analysis between phylum-level gut microbiota and environmental factors. **B** Correlation analysis between genus-level gut microbiota and environmental factors. **C** Relationship between gut microbiota composition and inter-population geographic distance. **D** Regression analysis between geographic distance and genetic distance among plateau zokor populations. (Lat: latitude; AP: annual precipitation; Long: longitude; Bio3: isothermality; Alt: altitude; Pm1: precipitation in January.)

The db-RDA analysis revealed a significant association between environmental factors and the gut microbiota composition of plateau zokor populations from different geographic locations (Supplementary Fig. 2). At both the phylum and genus levels, environmental variables such as latitude (Lat), altitude (Alt), annual precipitation (AP), isothermality (Bio3), and average monthly temperature (AT) were aligned with the directions of variation in gut microbial community structure in the ordination space, indicating a close relationship between these factors and community composition. Correlation analysis further revealed specific associations between environmental factors and microbial taxa. At the phylum level (Fig. 5A), dominant phyla including *Candidatus Saccharibacteria*, *Proteobacteria*, *Bacteroidota*, and an *unclassified genus from the Lachnospiraceae family* showed significant correlations with latitude, altitude, and precipitation-related variables. At the genus level (Fig. 5B), gut microbial taxa such as *Desulfovibrio*, *Ruminococcus*, *Bacteroides*, *Muribaculaceae*, and an *unclassified genus from the Lachnospiraceae family* exhibited significant positive or negative correlations with various environmental factors. Overall, different environmental factors displayed distinct correlation patterns with gut microbiota structure at the phylum and genus levels.

To evaluate the effect of geographic distance on the similarity of gut microbial communities among different plateau zokor populations, the Bray-Curtis similarity index was used to calculate the pairwise community similarity, and a scatter plot of community similarity against geographic distance was generated (Fig. 5C). The results showed that gut microbial community similarity among different plateau zokor populations decreased with increasing geographic distance (R² = 0.001, *P* = 0.020), indicating that geographic distance partially drives the differences in gut microbiota among these populations.

Pairwise analysis of genetic distances among different plateau zokor populations indicated significant genetic differentiation between populations. Overall, geographically closer populations (e.g., HL–GH, HL–MD, GH–GC) exhibited lower genetic differentiation, whereas populations separated by greater distances or influenced by environmental barriers (e.g., CD–QL, CD–GH) showed higher genetic differentiation (Supplementary Table 5). Genotyping was performed at 10 highly polymorphic SSR loci, and the summary statistics for each locus were as follows: average number of alleles (Na) = 3.73, average effective number of alleles (Ne) = 2.52, average Shannon’s information index (I) = 0.93, average observed heterozygosity (Ho) = 0.46, and average expected heterozygosity (He) = 0.49 (Supplementary Table 6). These results indicate that the selected SSR loci exhibit high polymorphism and contain substantial genetic information.

The relationship between geographic distance and genetic distance among different plateau zokor populations was assessed using Mantel tests and linear regression analysis (Fig. 5D). The results showed a significant positive linear correlation between genetic distance and geographic distance. In addition, based on geographic distance, the pairwise comparisons were divided into two groups: the short-distance group (geographic distance < 300 km, shown as red circles) and the long-distance group (geographic distance ≥ 300 km, shown as blue triangles). Both groups exhibited an increasing trend in genetic distance with greater geographic distance, and the genetic distances in the long-distance group were generally higher than those in the short-distance group. This finding suggests that a higher degree of geographic isolation corresponds to a greater level of genetic differentiation among plateau zokor populations.

## Discussion

### The gut microbiota of different plateau zokor populations showed significant differences

The gut microbiota in animals is a micro-ecosystem composed of various microorganisms, including archaea, bacteria, fungi, viruses, and bacteriophages, which play key roles in host physiological functions such as nutrient utilization, immune regulation, and energy metabolism [22, 23]. In this study, metagenomic sequencing was used to systematically analyze the gut microbial communities of different plateau zokor populations. The results showed that at the phylum level, the dominant gut microbial phyla were *Firmicutes* and *Bacteroidetes*, which is highly consistent with the core gut microbiota composition of typical herbivorous animals, such as Gansu zokors and New Zealand white rabbits [24, 25]. *Firmicutes* participate in the degradation of cellulose and hemicellulose, promoting host energy absorption and short-chain fatty acid (SCFA) metabolism, while also maintaining intestinal homeostasis by regulating immune function. These roles are particularly important in the cold, hypoxic, and high-energy-demand environments of the plateau [26, 27]. *Bacteroidetes* mainly assist the host in decomposing carbohydrates and proteins, thereby improving nutrient utilization efficiency [28, 29]. In addition, when the abundance of *Firmicutes* exceeds that of *Bacteroidetes*, energy extraction from food can be enhanced, promoting fat accumulation and body mass gain [30]. In this study, some high-altitude populations exhibited a significantly increased *Firmicutes*/*Bacteroidetes* (F/B) ratio, which may enhance intestinal energy absorption and fat storage, thereby supporting larger body size and adaptation to the plateau environment [31, 32].

At the genus level, the dominant gut bacterial genera in plateau zokors were the *unclassified genus of the Lachnospiraceae family*, *Ruminococcus*, and *Desulfovibrio*. These microbes play important roles in host energy metabolism and immune homeostasis [33–35]. The *unclassified genus of Lachnospiraceae* helps maintain gut barrier integrity and immune balance through SCFA production, while *Ruminococcus* secretes cellulases and hemicellulases to enhance plant fiber degradation and also produces anti-inflammatory SCFAs [36–38]. In the high-altitude environment, plateau zokors face the dual pressures of insufficient energy intake and hypoxia, making the stability and function of their gut microbiota essential for nutrient breakdown, energy acquisition, and overall physiological homeostasis. In addition, this study detected several methanogenic archaea (methanogens) in the gut microbiota of plateau zokors, including Methanobrevibacter, Methanosphaera, and related taxa within *Euryarchaeota*, suggesting that active methanogenesis may occur in their intestines. As an important greenhouse gas, methane production may not only influence local greenhouse gas fluxes but may also enter the soil environment through animal feces, thereby indirectly regulating soil carbon cycling processes [39]. In alpine grassland ecosystems, plateau zokors continuously introduce gut microorganisms and their metabolic products into the soil through activities such as plant consumption, burrowing, and defecation. This process may stimulate the metabolic activity of soil microbial communities, thereby influencing soil carbon and nitrogen cycling and potentially affecting the carbon dynamics and greenhouse gas emission patterns of regional grassland ecosystems. Therefore, future studies are needed to further quantify the actual contribution of these gut microbial communities to soil greenhouse gas fluxes and to evaluate their potential ecological roles in grassland ecosystem management and responses to climate change.

In this study, the alpha diversity indices revealed significant differences in the richness and diversity of gut microbiota among different plateau zokor populations, suggesting ecological differentiation in the structural diversity of gut microbial communities among these geographic populations. The gut microbial community often changes in response to variations in the host’s genotype, physiological status, environmental factors, and dietary composition [26, 40, 41]. Differences in the living environments of plateau zokor populations may lead to variations in the diversity and richness of their gut microbiota. In the beta diversity analysis, the results of NMDS and PCoA analyses were consistent, showing that individuals from the same plateau zokor population clustered together, while significant differences existed in the gut microbiota among the nine geographically distinct populations. This indicates that geographic origin is an important factor influencing the composition of the gut microbiota in plateau zokors. Notably, the HN population consistently exhibited the most pronounced differentiation from other populations across β-diversity analyses. This pattern may be closely associated with its distinctive ecological conditions and long-term, persistent anthropogenic disturbances. The plateau zokor population density in the HN region is relatively high and has been subjected to sustained control measures [42]. Previous studies have shown that rodent control practices in this area can significantly increase aboveground plant biomass, with particularly strong positive effects on monocotyledonous forage species [43]. Such shifts in vegetation structure may alter the relative intake of carbohydrates and cellulose in the host diet, thereby exerting selective pressures on gut microbial taxa involved in fiber degradation and energy metabolism [44–48]. In addition, the HN sampling sites are characterized by widespread human activity and grazing disturbance. Grazing by livestock can substantially influence grassland ecosystems by modifying plant community composition and increasing surface soil compaction [49, 50]. Plateau zokor population densities are significantly higher in seasonally grazed and freely grazed areas than in grazing-excluded sites [51]. Previous research has demonstrated that the abundance of gut microbiota in plateau zokors is closely associated with soil bulk density and compaction [52]. Furthermore, this study revealed that the HN population exhibits a higher level of host genetic differentiation compared with other geographic populations, which may further amplify the distinctiveness of its gut microbiota. A growing body of animal studies indicates that host genetic background is a key intrinsic factor shaping gut microbial community structure. For example, in wild macaques (*Macaca* spp.), genetic differentiation among geographically distinct populations has been shown to be significantly associated with differences in gut microbiota composition [53]. Taken together, the pronounced gut microbiota differentiation observed in the HN population is likely driven by the combined effects of host genetic divergence and its unique ecological environment.

### Geographic distance, environmental factors, and genetic differentiation jointly drive the gut microbiota structure of plateau zokor populations

The composition and function of the gut microbiota in plateau zokors are jointly influenced by multiple environmental factors, including temperature, precipitation, and the nutritional composition of local vegetation [54]. The results of this study indicate that geographic distance, environmental factors, and host genetics jointly drive the differentiation of gut microbial communities among different geographical populations of plateau zokors. Notably, populations separated by greater spatial distances (such as those between eastern and western sampling sites) exhibited more pronounced differences in community structure. Previous studies have shown that gut microbial communities exhibit a significant distance-decay pattern [55]. The macro biogeographic pattern suggests that ecological processes operating across spatial scales play an important role in shaping the structure of host gut microbial communities, with differences in community composition increasing progressively with geographic distance [56, 57]. Moeller et al. [44] found that the compositional similarity of gut microbial communities among mammals decreases exponentially with increasing geographical distance between hosts. Different environmental conditions can significantly influence the interactions between symbiotic microorganisms and their hosts in the gut, thereby altering the composition and distribution patterns of intestinal microbiota. This variation may be attributed to differences in geographical environmental factors and dietary resources [58, 59]. Geographical isolation is also an important factor contributing to microbial community differentiation. The genetic divergence caused by geographic barriers often has a significant impact on the structure of the gut microbiota [60]. The differences in the dominant bacterial phyla within the animal gut are significantly correlated with the host’s genetic background [61]. In this study, Mantel tests confirmed that both the geographic distance versus differentiation index and geographic distance versus genetic distance among plateau zokor populations were significantly positively correlated. Due to the large geographic distances, gene flow between plateau zokor populations is limited, leading to significant differentiation in their gut microbial community composition. This is consistent with the findings of Cai Zhenyuan et al. [13] who studied geographic isolation in plateau zokor populations using partial mitochondrial DNA sequences and found that genetic differentiation in plateau zokors was highly significantly positively correlated with geographic distance. On one hand, the dispersal and migration abilities of plateau zokors are limited, and geographic barriers promote genetic drift by restricting gene flow; on the other hand, heterogeneous selective pressures drive adaptive evolution. The combined effect of these factors accelerates the process of genetic differentiation among populations [62]. Environmental factors play a crucial role in shaping gut microbial community structure. In this study, analysis of gut microbiota across different geographic populations of plateau zokors showed that environmental variables, including latitude, altitude, annual precipitation, isothermality, and mean monthly temperature, were significantly associated with gut microbial composition at both the phylum and genus levels. This suggests that environmental differences exert a selective effect on the gut microbial community structure of different plateau zokor populations, supporting the “environmental isolation” hypothesis. In other words, microbial community differentiation can be driven solely by environmental variation, rather than relying entirely on increasing geographic distance [63]. The differences in habitat among plateau zokor populations may indirectly shape gut microbial community structure by influencing vegetation types, food resource composition, and host physiological status [64, 65]. Similar environment-driven patterns of gut microbiota differentiation have been observed in various wild mammals and other high-altitude species. On the Qinghai-Tibet Plateau, the gut microbial community structure of ungulates, such as Tibetan antelope, yak, and plateau pika, changes significantly along altitude and climatic gradients [66, 67]. Plateau zokors live in underground burrow environments for extended periods, facing multiple ecological pressures such as low temperature, hypoxia, and seasonal fluctuations in resource availability. In this context, the gut microbiota plays a particularly important role in energy acquisition and metabolic regulation. *Ruminococcus*, *Bacteroides*, and the unclassified genus of *Lachnospiraceae* are key players in the degradation of complex polysaccharides and the synthesis of short-chain fatty acids (SCFAs), while *Muribaculaceae* is considered an important group for plant polysaccharide utilization in the gut microbiota of rodents. Many genera significantly associated with environmental factors are involved in carbohydrate fermentation and SCFA production. These metabolic products not only provide additional energy for the host but also participate in the regulation of host immune function and energy homeostasis, thereby enhancing host survival under extreme environmental conditions [68, 69].

### The gut microbial functions of different plateau zokor populations exhibit significant differences

KEGG functional annotation revealed that the gut microbiota of plateau zokors exhibits relatively high abundance in carbohydrate metabolism and amino acid metabolism, indicating that metabolic functions occupy a central role in their gut microbial communities. This pattern is highly consistent with the gut functional profiles observed in other herbivorous animals [70]. Under the harsh conditions of the plateau and the relative scarcity of food resources, plateau zokors rely on their gut microbiota to enhance food digestion efficiency and energy acquisition, thereby meeting the high energy demands required for survival in high-altitude environments [71]. The gut microbial communities of plateau zokors from different geographic populations exhibit functional differences, which may be closely related to environmental factors and geographic distance. For example, variables such as altitude and annual precipitation have significant impacts on the gut microbiota of plateau zokors. Environmental differences indirectly shape microbial functional structures by influencing vegetation types, food resource composition, and host physiological status [64, 65].

In the analysis of carbohydrate-active enzymes (CAZy), enzymes related to the degradation of cellulose, hemicellulose, lignin, and other plant fibers showed significant variation among gut microbiota from different populations. Adjustments in microbial composition and enzyme systems not only facilitate efficient food breakdown but also provide the host with more readily available energy [72], and may enhance the host’s ability to cope with environmental changes by modulating physiological status [73]. Studies have shown that in herbivorous animals, enzymes involved in cellulose degradation are significantly enriched in the gut microbiota, promoting efficient energy extraction from high-fiber plant tissues [74]. In plateau zokors, carbohydrate-active enzymes are primarily composed of GTs (glycosyltransferases) and GHs (glycoside hydrolases). Among them, the GT2 family of GTs participates in various biosynthetic processes by catalyzing glycosyl transfer reactions, while GT4 is involved in glycosidic bond formation, playing important roles in energy storage, cell signaling, and cell wall synthesis. GH13 mainly functions in the hydrolysis of starch and other α-glycosidic bonds [75, 76]. In mammals, cellulose degradation largely depends on gut symbiotic microorganisms, and carbohydrate-active enzymes play a key role in carbohydrate metabolism by catalyzing the degradation, modification, and synthesis of glycosidic bonds [77].

Plateau zokors primarily feed on plant roots and rhizomes that are rich in fiber and sugars. Their gut microbiota is highly adapted to this high-fiber diet, enabling efficient degradation and utilization of plant fibers to provide energy and essential nutrients for the host. For herbivorous animals, the metabolic capacity of the gut microbiota is critical for plant fiber degradation and nutrient absorption. Functional differences in the gut microbiota among different geographic populations of plateau zokors may confer adaptive advantages, enhancing host adaptability and survival in diverse environmental conditions.

## Conclusion

The core gut microbiota of plateau zokors from different geographic populations is primarily composed of *Firmicutes*, *Bacteroidetes*, and *Proteobacteria*. Significant differences were observed in community composition, α-diversity, and functional potential, particularly in carbohydrate metabolism and carbohydrate-active enzymes involved in the degradation of cellulose, hemicellulose, and lignin. Environmental factors such as latitude, annual precipitation, and altitude, as well as geographic distance, had significant impacts on gut microbial community structure, with genetic distance among populations increasing with geographic distance. Overall, the differentiation of gut microbiota in plateau zokors appears to result from the combined effects of geographic isolation, genetic divergence, and environmental heterogeneity, highlighting the critical role of gut microbiota in host ecological adaptation and providing a reference for the scientific management of grassland rodent populations.

This study did not conduct systematic surveys of the vegetation composition or food sources at each sampling site, and therefore dietary-related variables could not be incorporated into the statistical models. Future studies could integrate vegetation surveys or fecal plant DNA metabarcoding to further investigate the relationship between host diet composition and gut microbial community functions, thereby providing a more comprehensive understanding of how environmental factors and diet influence gut microbiome functionality.

## Supplementary Information

Supplementary Material 1.

Supplementary Material 2.

## Data Availability

The datasets generated in this study have been deposited in the Genome Sequence Archive (GSA) of the National Genomics Data Center (NGDC) under accession number CRA033318. The data are publicly available at: https://ngdc.cncb.ac.cn/gsa/s/QF6s5ZvC.
