# Supplementary material for: Metagenomics reveals gut microbial differences and ecological adaptation in plateau zokor (Eospalax baileyi) populations
Source: BMC Microbiol. 2026 Apr 20;26:519. doi: 10.1186/s12866-026-05069-6 (PMC13231566; doi:10.1186/s12866-026-05069-6)
Supplement: Supplementary file 1 — Supplementary Material 1. [file 12866_2026_5069_MOESM1_ESM.zip › Supplementary Material 1/Supplementary table S1 The information of sampling sites of plateau zokor..docx]

**Supplementary table S1:** The information of sampling sites of plateau zokor.

| Sampling site | East longitude | North latitude | Altitude（m） | The number of male zokors |
| --- | --- | --- | --- | --- |
| DT | 101°14'34.8" | 37°08'31.2" | 3,127 | 10 |
| GH | 100°10'26.4" | 36°35'24.0" | 3,254 | 10 |
| HZ | 102°07'08.4" | 36°53'42.0" | 3,014 | 9 |
| QL | 100°20'06.0" | 38°07'30.0" | 3,032 | 14 |
| MD | 98°17'27.6" | 34°56'42.0" | 4,460 | 13 |
| CD | 97°13'19.2" | 33°29'56.4" | 4,360 | 16 |
| HL | 102°17'13.2" | 36°11'02.4" | 3,141 | 17 |
| HN | 101°33'46.8" | 34°46'01.2" | 3,565 | 18 |
| GC | 100°25'55.2" | 37°34'44.4" | 3,554 | 6 |
