# Supplementary material for: Metagenomics reveals gut microbial differences and ecological adaptation in plateau zokor (Eospalax baileyi) populations
Source: BMC Microbiol. 2026 Apr 20;26:519. doi: 10.1186/s12866-026-05069-6 (PMC13231566; doi:10.1186/s12866-026-05069-6)
Supplement: Supplementary file 1 — Supplementary Material 1. [file 12866_2026_5069_MOESM1_ESM.zip › Supplementary Material 1/Supplementary table S2 Sample sequencing information..docx]

**Supplementary table S2:** Sample sequencing information.

| Population | raw reads | clean reads | GC/% | Q20/% | Q30/% |
| --- | --- | --- | --- | --- | --- |
| DT | 94 711 644.00 | 93 708 502.00 | 47.15 | 98.04 | 94.30 |
| GH | 91 806 945.80 | 90 712 220.00 | 47.18 | 97.97 | 94.41 |
| HZ | 98 701 825.78 | 97 525 328.44 | 47.11 | 98.08 | 94.31 |
| QL | 95 671 958.29 | 94 583 716.00 | 46.68 | 98.16 | 94.48 |
| MD | 90 108 101.54 | 89 154 706.92 | 47.21 | 97.98 | 94.05 |
| CD | 93 787 964.63 | 92 772 358.50 | 47.45 | 98.08 | 94.30 |
| HL | 98 625 033.41 | 97 365 158.35 | 48.04 | 98.01 | 94.16 |
| HN | 95 446 809.67 | 94 399 622.78 | 48.04 | 98.12 | 94.41 |
| GC | 92 371 415.67 | 91 367 348.67 | 48.43 | 98.17 | 94.55 |

Q20 and Q30 refer to the percentage of total bases in clean reads for bases with mass values greater than or equal to 20 and 30, respectively.
