# Supplementary material for: Metagenomics reveals gut microbial differences and ecological adaptation in plateau zokor (Eospalax baileyi) populations
Source: BMC Microbiol. 2026 Apr 20;26:519. doi: 10.1186/s12866-026-05069-6 (PMC13231566; doi:10.1186/s12866-026-05069-6)
Supplement: Supplementary file 1 — Supplementary Material 1. [file 12866_2026_5069_MOESM1_ESM.zip › Supplementary Material 1/Supplementary table S3 Statistical table of assembly results..docx]

**Supplementary table S3:** Statistical table of assembly results.

| Population | contigs | contigs bases/bp | N50/bp | N90/bp | Max/bp) | Min/bp | ORFs |
| --- | --- | --- | --- | --- | --- | --- | --- |
| DT | 942 795.00 | 701 084 072.90 | 801.20 | 364.50 | 332 517.50 | 300 | 1 309 317.80 |
| GH | 917 266.70 | 763 298 271.30 | 983.40 | 379.30 | 298 907.40 | 300 | 1 336 292.70 |
| HZ | 1 173 714.00 | 887 303 659.70 | 838.708 | 367.67 | 328 398.67 | 300 | 1 620 076.00 |
| QL | 808 994.36 | 670 313 951.80 | 1 259.86 | 384.57 | 319 923.93 | 300 | 1 176 484.93 |
| MD | 855 664.69 | 714 304 654.90 | 981.54 | 377.54 | 341 863.00 | 300 | 1 240 457.54 |
| CD | 913 657.81 | 727 653 187.60 | 906.13 | 373.56 | 368 151.25 | 300 | 1 304 712.50 |
| HL | 1 244 905.65 | 923 771 568.60 | 807.29 | 365.00 | 311 523.53 | 300 | 1 719 523.54 |
| HN | 1 267 970.50 | 916 418 468.30 | 771.22 | 361.56 | 363 173.67 | 300 | 1 701 807.33 |
| GC | 1 168 873.67 | 856 686 422.50 | 785.50 | 364.17 | 492 691.33 | 300 | 1 598 365.50 |

N50 (N90) refers to the sequence of contigs sorted by length, and the length values of each sequence scanned one by one from large to small are accumulated. When the accumulated value exceeds 50 % (90 %) of the total length of all sequences for the first time, the sequence scanned at this time; max refers to the sequence length of the longest contigs; min refers to the sequence length of the shortest contigs.
