# Supplementary material for: Metagenomics reveals gut microbial differences and ecological adaptation in plateau zokor (Eospalax baileyi) populations
Source: BMC Microbiol. 2026 Apr 20;26:519. doi: 10.1186/s12866-026-05069-6 (PMC13231566; doi:10.1186/s12866-026-05069-6)
Supplement: Supplementary file 1 — Supplementary Material 1. [file 12866_2026_5069_MOESM1_ESM.zip › Supplementary Material 1/Supplementary table S4 Top 20 functional categories by relative abundance at KEGG Level 2..docx]

**Supplementary table S4:** Top 20 functional categories by relative abundance at KEGG Level 2.

| KEGG pathway Level2 | Mean（%） | SD（%） |
| --- | --- | --- |
| Global and overview maps | 40.51% | 0.23% |
| Carbohydrate metabolism | 9.18% | 0.16% |
| Amino acid metabolism | 5.80% | 0.04% |
| Replication and repair | 4.20% | 0.08% |
| Energy metabolism | 3.55% | 0.06% |
| Membrane transport | 3.42% | 0.12% |
| Metabolism of cofactors and vitamins | 3.16% | 0.09% |
| Translation | 2.86% | 0.09% |
| Signal transduction | 2.79% | 0.11% |
| Glycan biosynthesis and metabolism | 2.74% | 0.08% |
| Nucleotide metabolism | 2.63% | 0.02% |
| Cellular community - prokaryotes | 2.10% | 0.08% |
| Cell motility | 1.79% | 0.13% |
| Lipid metabolism | 1.67% | 0.04% |
| Folding, sorting and degradation | 1.51% | 0.03% |
| Biosynthesis of other secondary metabolites | 1.47% | 0.03% |
| Infectious disease: bacterial | 1.16% | 0.05% |
| Metabolism of other amino acids | 1.15% | 0.02% |
| Drug resistance: antimicrobial | 1.09% | 0.02% |
| Cell growth and death | 0.93% | 0.02% |
| others | 6.32% | 0.05% |

KEGG pathway Level 2 was the name of metabolic pathway. Mean (%) is the percentage value of the mean; Sd (%) is the percentage of standard deviation.
