# Supplementary material for: Metagenomics reveals gut microbial differences and ecological adaptation in plateau zokor (Eospalax baileyi) populations
Source: BMC Microbiol. 2026 Apr 20;26:519. doi: 10.1186/s12866-026-05069-6 (PMC13231566; doi:10.1186/s12866-026-05069-6)
Supplement: Supplementary file 1 — Supplementary Material 1. [file 12866_2026_5069_MOESM1_ESM.zip › Supplementary Material 1/Supplementary table S5 Genetic distance (Fst) of plateau zokor populations..docx]

**Supplementary table S5:** Genetic distance (*Fst*) of plateau zokor populations.

| Population | HN | HL | CD | HZ | GH | MD | DT | QL | GC |
| --- | --- | --- | --- | --- | --- | --- | --- | --- | --- |
| HN | 0.000 | 0.204 | 0.355 | 0.222 | 0.278 | 0.243 | 0.208 | 0.279 | 0.253 |
| HL | 0.204 | 0.000 | 0.364 | 0.175 | 0.112 | 0.087 | 0.142 | 0.105 | 0.118 |
| CD | 0.355 | 0.364 | 0.000 | 0.345 | 0.376 | 0.397 | 0.324 | 0.403 | 0.381 |
| HZ | 0.222 | 0.175 | 0.345 | 0.000 | 0.230 | 0.205 | 0.148 | 0.241 | 0.180 |
| GH | 0.278 | 0.112 | 0.376 | 0.230 | 0.000 | 0.079 | 0.163 | 0.140 | 0.167 |
| MD | 0.243 | 0.087 | 0.397 | 0.205 | 0.079 | 0.000 | 0.141 | 0.126 | 0.154 |
| DT | 0.208 | 0.142 | 0.324 | 0.148 | 0.163 | 0.141 | 0.000 | 0.158 | 0.159 |
| QL | 0.279 | 0.105 | 0.403 | 0.241 | 0.140 | 0.126 | 0.158 | 0.000 | 0.083 |
| GC | 0.253 | 0.118 | 0.381 | 0.180 | 0.167 | 0.154 | 0.159 | 0.083 | 0.000 |
