# Supplementary material for: Metagenomics reveals gut microbial differences and ecological adaptation in plateau zokor (Eospalax baileyi) populations
Source: BMC Microbiol. 2026 Apr 20;26:519. doi: 10.1186/s12866-026-05069-6 (PMC13231566; doi:10.1186/s12866-026-05069-6)
Supplement: Supplementary file 1 — Supplementary Material 1. [file 12866_2026_5069_MOESM1_ESM.zip › Supplementary Material 1/Supplementary table S6 Polymorphism information of SSR primer.docx]

**Supplementary table S6:** Polymorphism information of SSR primer

| Locus | Na | Ne | I | Ho | He |
| --- | --- | --- | --- | --- | --- |
| L077 | 2.222 | 1.543 | 0.505 | 0.025 | 0.308 |
| L798 | 2.111 | 1.504 | 0.480 | 0.431 | 0.301 |
| L262 | 2.000 | 1.447 | 0.378 | 0.235 | 0.222 |
| L447 | 5.444 | 3.368 | 1.344 | 0.706 | 0.647 |
| L525 | 4.556 | 2.927 | 1.140 | 0.505 | 0.578 |
| L234 | 6.333 | 3.937 | 1.506 | 0.805 | 0.725 |
| L116 | 4.222 | 3.062 | 1.211 | 0.693 | 0.656 |
| L331 | 3.444 | 2.621 | 1.012 | 0.276 | 0.573 |
| L409 | 4.778 | 3.389 | 1.288 | 0.710 | 0.664 |
| L280 | 2.222 | 1.405 | 0.399 | 0.240 | 0.230 |
| Mean | 3.73 | 2.52 | 0.93 | 0.46 | 0.49 |
