# Supplementary material for: Metagenomics reveals gut microbial differences and ecological adaptation in plateau zokor (Eospalax baileyi) populations
Source: BMC Microbiol. 2026 Apr 20;26:519. doi: 10.1186/s12866-026-05069-6 (PMC13231566; doi:10.1186/s12866-026-05069-6)
Supplement: Supplementary file 2 — Supplementary Material 2. [file 12866_2026_5069_MOESM2_ESM.zip › Supplementary Material 2/Supplementary Fig. 1 Geographic locations of plateau zokor sampling sites..docx]

**Supplementary Figure 1：**


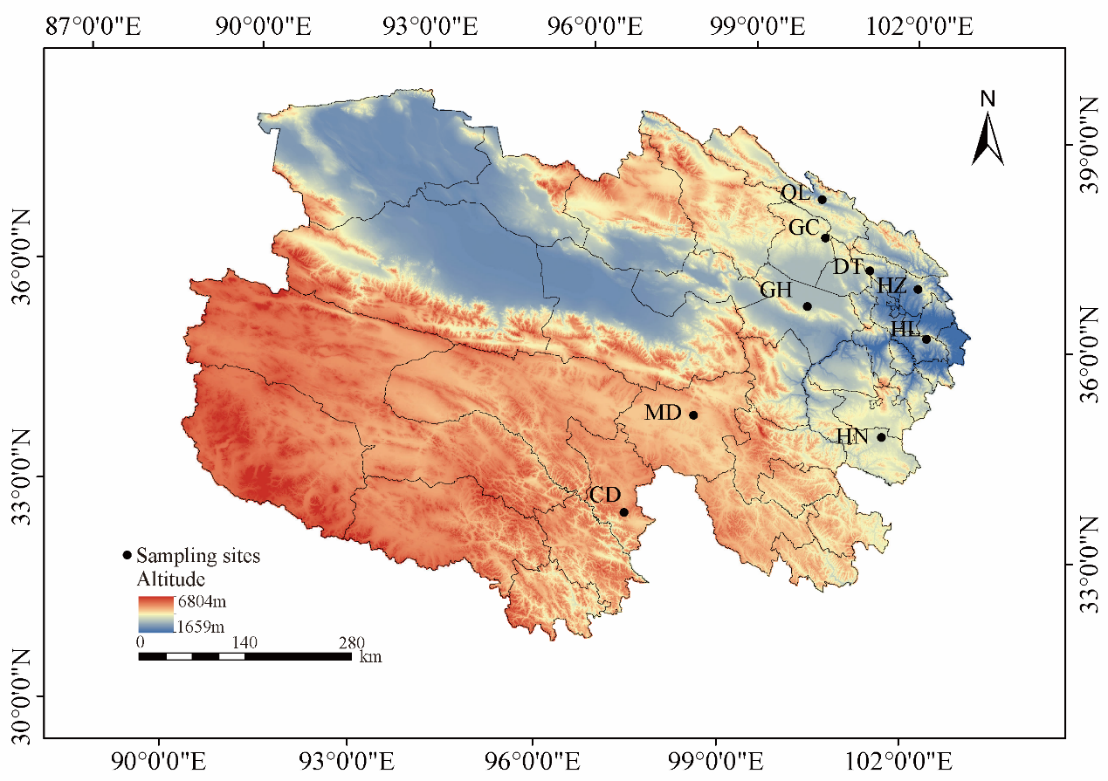


**Supplementary Fig 1** Geographic locations of plateau zokor sampling sites.
