# Supplementary material for: Metagenomics reveals gut microbial differences and ecological adaptation in plateau zokor (Eospalax baileyi) populations
Source: BMC Microbiol. 2026 Apr 20;26:519. doi: 10.1186/s12866-026-05069-6 (PMC13231566; doi:10.1186/s12866-026-05069-6)
Supplement: Supplementary file 2 — Supplementary Material 2. [file 12866_2026_5069_MOESM2_ESM.zip › Supplementary Material 2/Supplementary Fig. 2 db-RDA analysis of gut microbiota composition at the phylum and genus levels in relation to environmental factors..docx]

**Supplementary Figure 2：**


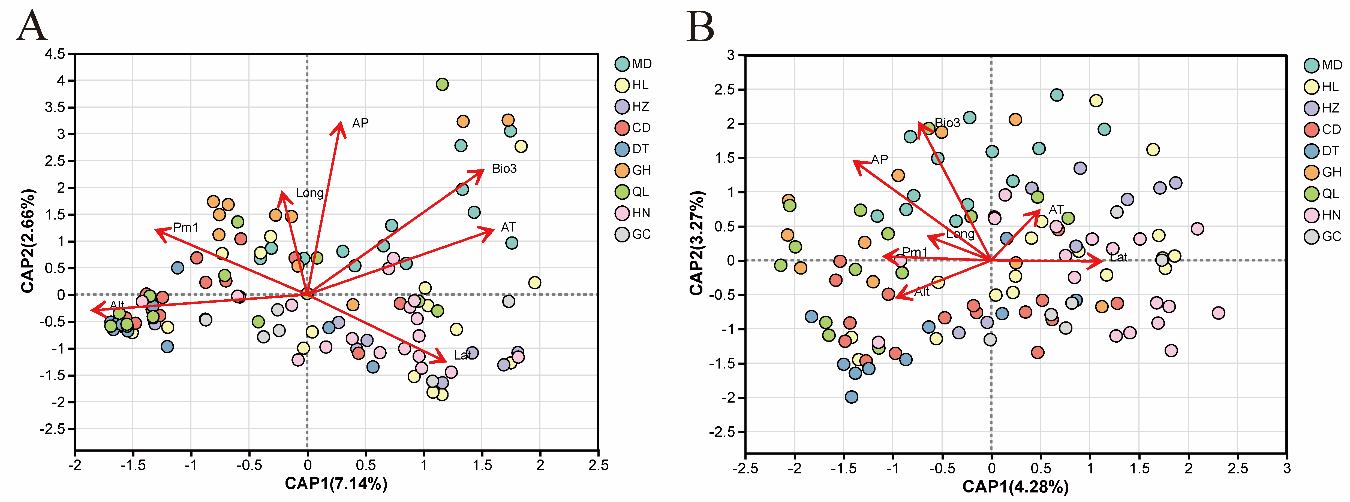


**Supplementary Figure 2** Redundancy analysis (RDA) of environmental factors and gut microbiota. (A) Phylum-level RDA based on Bray–Curtis distances. (B) Genus-level RDA based on Bray–Curtis distances. (Lat: latitude; AP: annual precipitation; Long: longitude; Bio3: isothermality; Alt: altitude; Pm1: precipitation in January.)
